# The COVID-19 impacts on bikeshare systems in small rural communities: Case study of bikeshare riders in Montgomery County, VA

**DOI:** 10.1371/journal.pone.0278207

**Published:** 2022-12-01

**Authors:** Mohammed Almannaa, Cat Woodson, Huthaifa Ashqar, Mohammed Elhenawy

**Affiliations:** 1 Department of Civil Engineering, College of Engineering, King Saud University, Riyadh, Saudi Arabia; 2 School of Public and International Affairs, Virginia Tech, Blacksburg, VA, United States of America; 3 Precision Systems, Inc. Arab American University, Washington, DC, United States of America; 4 Centre for Accident Research and Road Safety, Queensland University of Technology, Brisbane, Australia; The University of Tokyo, JAPAN

## Abstract

The shared and micro-mobility industry (ride sharing and hailing, carpooling, bike and e-scooter shares) saw direct and almost immediate impacts from COVID-19 restrictions, orders and recommendations from local governments and authorities. However, the severity of that impact differed greatly depending on variables such as different government guidelines, operating policies, system resiliency, geography and user profiles. This study investigated the impacts of the pandemic regarding bike-share travel behavior in Montgomery County, VA. We used bike-usage dataset covering two small towns in Montgomery county, namely: Blacksburg and Christiansburg, including Virginia Tech campus. The dataset used covers the period of Jan 2019—Dec 2021 with more than 14,555 trips and 5,154 active users. Findings indicated that a bikeshare user’s average trip distance and duration increased in 2020 (compared to 2019) from 2+ miles to 4+ and from half an hour to about an hour. While there was a slight drop in 2021, bikeshare users continued to travel farther distances and spend more time on the bikes than pre-COVID trips. When those averages were unpacked to compare weekday trips to weekend trips, a few interesting trip patterns were observed. Unsurprisingly, more trips still took place on the weekends (increasing from 2x as many trips to 4x as many trips than the weekday). These findings could help to better understand traveler’s choices and behavior when encountering future pandemics.

## Introduction

Over the past few years, the U.S and others have been trying to recover from the devastating impacts of the COVID-19 pandemic. Some communities were still recovering from the 2008 recession before this global disaster hit. For those living in more rural areas and/or in smaller, more isolated towns, the pandemic posed greater difficulties to daily living and quality of life. In general, transportation options for most rural areas are already limited, with residents almost exclusively needing to rely on private vehicles to travel. However, those who do not own or have access to a car, must find other modes of transportation to meet their travel needs. The impacts of the pandemic further showcased existing transportation disparities in these communities.

Still, limited research is available on the unique challenges rural regions face concerning transportation and health. Transportation studies tend to look at physical activity as it relates to utilitarian use or non-motorized travel, while health studies that include walking and biking tend to focus more on leisure or recreational use. Even when they do cross each other, data collection is usually more focused on vehicular travel with health relationships centering around traffic-related pedestrian/bicyclist injuries, fatalities, and/or air pollution concerns; those studies may still overlook and under-count non-motorized travel/trips.

Some of the key barriers preventing more equitable active transportation in rural communities are infrastructure, geography, funding, accessibility, political support, public awareness, and finally socio-demographics [1]. CityStudio Vancouver defines equitable active transportation as “…improving the quality of life of community members by increasing their opportunities to access life-enriching activities through high-quality routes and active transportation options.”. Securing sustainable funding sources, developing and maintaining infrastructure that supports walking and biking and enables safe access for all users, are key points for increasing active transportation equity in rural areas. Conducting more research on the links between active transportation and health could benefit rural communities in gaining the necessary means to improve their infrastructure, so more residents would feel encouraged and safe to use active modes to get around. Thus, it is important for governments to enable, encourage, and improve better access to active transportation, with policies like “The Transportation Prescription Healthy and Equitable Transportation Policy”. Implementing policies such as Complete Streets, is particularly beneficial for more vulnerable populations such as low-income families, children, and older adults.

Although bikeshare has been around since the 1960s, it did not really take off in the United States until 2008. Now, however, bikeshare systems are an essential element of communities all around North America. This active means of transportation has become popular across the nation more than ten years later. However, not all American localities have been able to benefit from this expanding transportation trend. Since urban areas were the first to use this mode, networks spanning metropolitan areas, cities, neighborhoods, employment hubs, and/or university campuses are more likely to have bikeshare. This means that residents in suburban and rural locations will less frequently have access to bikeshare services. In particular, rural communities present a unique challenge to bikeshare operators e.g. further delays in providing and maintaining the same level of multimodal integration most urban cities take advantage of. Bikeshare programs are also often implemented in a community as a way to get people out of their cars and into more sustainable modes of transportation. Unfortunately, this goal also excludes the needs of residents who are already car-less. Considering more other micro-mobility options in rural areas could be a way to bridge some of those existing transportation gaps.

Bikeshare systems operate under different characteristics in their implemented area depending on the size of the city or town. However, as seen in the 2009 National Household Travel Survey, 37% of the trips rural residents take are less than 3 miles [2]. Bikeshare programs are a unique option that can provide users a more accessible alternative to cars that are also active. Planners, developers and other decision makers have an opportunity to implement these programs to address some of the transportation and health inequities their communities face. Policy makers can help by supporting funding opportunities and implementing legislation that promotes active modes of transportation.

During the height of the pandemic, many bikeshare systems had to make the tough decision to suspend service or fully shutdown due to health concerns. Approximately 14% of bikeshare services were suspended during the pandemic. Fortunately, 75% of those suspended systems were successfully able to reopen by the end of 2020 [3–7]. Despite the statewide lockdown orders and restrictions in place, the bikeshare system in Montgomery County area remained fully operational, maintaining its pre-COVID service and bike fleet availability standards. This makes it possible to understand travelers’ mobility choices during COVID-19 pandemic.

This research effort aims mainly at studying the effect of COVID-19 on bike share usage in small rural community, namely: Montgomery County area. Thus, spatial and temporal analysis of trips made from 2019 to 2021, covering three periods: before, during-, and post-pandemic, were extracted and investigated. Findings would help in identifying how trip patterns and riders’ behavior have evolved in these three periods and thus operating agencies and policy makers can better understand the rider’s behavior during the future pandemics.

## Review of existing literature

As the use and acceptance of bikeshare becomes more evident, planners and developers have an opportunity to take advantage of this trend to address some of the transportation challenges rural communities face, providing more equitable transportation to residents and visitors. The spread of coronavirus (COVID-19) pandemic has globally impacted mobility including bikeshare systems. This impact was influenced by the adopted non-pharmacological control measures including social distancing, lockdowns, and remote style of working. However, as the debate over the future of transportation continues in the midst of the COVID-19 pandemic as a deepening global crisis, micro-mobility seems to be not spared by the quick and disrupting changes that arose from the pandemic that swept the world. Previous studies have investigated the short-term effect of COVID-19 pandemic on bike-sharing systems mobility in urban areas using either self-reported surveys or real-data.

An online survey in Sicily of Southern Italy investigated the influence of the COVID-19 pandemic on road users’ perceptions, needs, and use of sustainable travel modes (i.e., public transport, walking, and cycling) using an online survey during the period from March to May 2020 [8]. Results suggested that women were less likely to walk during the pandemic than men. Participants were more likely to resume remote work even after the second phase in order to reduce their daily travel needs and keep their isolation. Along with the adopted sustainability policies of the European cities, participants have expressed a positive opinion on the use of micromobility during pandemic situations [8].

Another survey study in Riyadh, Saudi Arabia, has explored the feasibility of launching an e-scooter sharing system as a new micromobility mode [9]. The study found that approximately half of the respondents believed that COVID-19 will not affect their willingness to ride e-scooters. Two types of logistic regression models were built. The outcomes of the models show that gender, age, and using ride-hailing services play an important role in respondents’ willingness to use e-scooter [9].

A study that synthesized knowledge on how the pandemic reshaped the relationship between cities and quality of life has found that the role of transport and land use, urban nature, public space, facilities and services, housing, and information and communications technology (ICT) in quality of life in cities was transformed during COVID-19 [10]. Access to many facilities and services; opportunities for walking and cycling; COVID-19-secure public transport; access to a car; urban blue or green space and access to nearby nature; easy access to open public space; living in a dwelling of sufficient size and quality; private or communal outdoor areas; and ICT infrastructure and systems have possibly helped to mitigate the negative impacts of COVID-19 on quality of life in urban areas [10].

Specifically, a study investigated the impact of COVID-19 on cycling using real-data as one of the modes that has enjoyed significant attention [11]. The study argued that cycling, in all its forms and variations including bike-sharing and e-bikes, emerged during the pandemic as a sustainable and economically feasible solution for relatively risk-free travel. It was also found that there is an increasing trend in the number of trips and traveled distances by bikes both for commuting and physical exercise/leisure purposes [11].

Another study–with the objective to provide an enhanced understanding of the impacts of COVID-19 crisis on travel behavior and shared mobility systems–has found that public transit and ride-hailing ridership have greatly decreased during the lockdowns but have increased once the lockdown was over [12]. The study argued that there are some changes that heralded as a consequence of the pandemic in the shared mobility space may remain long after the pandemic no longer remains a threat including stricter hygiene and cleaning standards, additional open space on the road for micromobility services, change in location decisions, vehicle occupancies, and vehicle miles traveled, change in the future of shared mobility services with newer business models, and renewed preference for ICT-enabled strategies, tele-activities, on-demand deliveries, and telecommuting [12].

A study in San Antonio (TX) investigated this impact by reviewing bike share system case studies in the United States and reports survey responses from bike share users. They found that there was an increase by 43% in using bike-sharing systems between the unemployed respondents due to the pandemic and a decrease of about 36% in ridership between employed respondents. They suggested that bike-sharing operators and policymakers should explore how to serve unemployed and low-income communities best and prepare for the equitable expansion of ridership following the pandemic [13]. Another study that assessed the impact of COVID-19 pandemic during the initial wave on biking in New York City (NYC), Boston, and Chicago [14]. They found that as the COVID-19 cases increased, these cities experienced a reduction in bikeshare trips with different rates between the three cities. The impact of COVID-19 was also experienced in decreasing bike trips and increasing the average duration of the trips during the pandemic. They also argued that NYC’s average trip duration was consistently less than that of Boston and Chicago, which could be due to its sprawl [14].

Lastly, a study utilized real-data has investigated the short-term changes in urban mobility, tropospheric air pollution, and fuel consumption in two major cities of Saudi Arabia, namely, Riyadh and Jeddah [15]. The study found that there was a significant reduction in urban mobility since the beginning of the first partial curfew in March 2020 compared to that in 2019, which caused the air pollutant levels and fuel consumption to be decreased [15].

As can be seen in the aforementioned research efforts, there were a focus on urban rather than rural areas in studying the effect of COVID-19 on bikeshare systems. The rural communities have different attributes and characteristics that need to be investigated separately and compared to other urban studies.

## Data and methods

### Definition of terms

For the purposes of this study, alternative transportation is defined as commuting by other modes than a car such as public transportation, walking and biking. Active Transportation is defined as commuting by human power such as walking and bicycling. Shared mobility is a broader term referring to the shared use of any vehicle, motorcycle, scooter, bicycle, and other travel modes. Share Micro-mobility continues to be an emerging transportation option, all shared-use fleets of small, fully, or partially human-powered vehicles such as bicycles, e-bicycles and e-scooters [2].

In the category of shared micro-mobility, bikesharing (also known as bikeshare or bike sharing system) often makes use of on-demand access to bicycles and/or e-bikes at a range of origin and destination locations. Public, closed campus, and peer-to-peer bikesharing systems are the three most common varieties. The most widespread bikesharing programs are public (with docked/stationed and dockless) ones that let anyone use a bike for a price. Bikes that can only be used by university associates or inside the campus boundaries are referred to as being on "closed campuses." Peer-to-peer bike sharing makes advantage of mobile location services and the social networks of current users and bike owners.

### Dataset—bikeshare in the New River Valley

**Fig 1:**
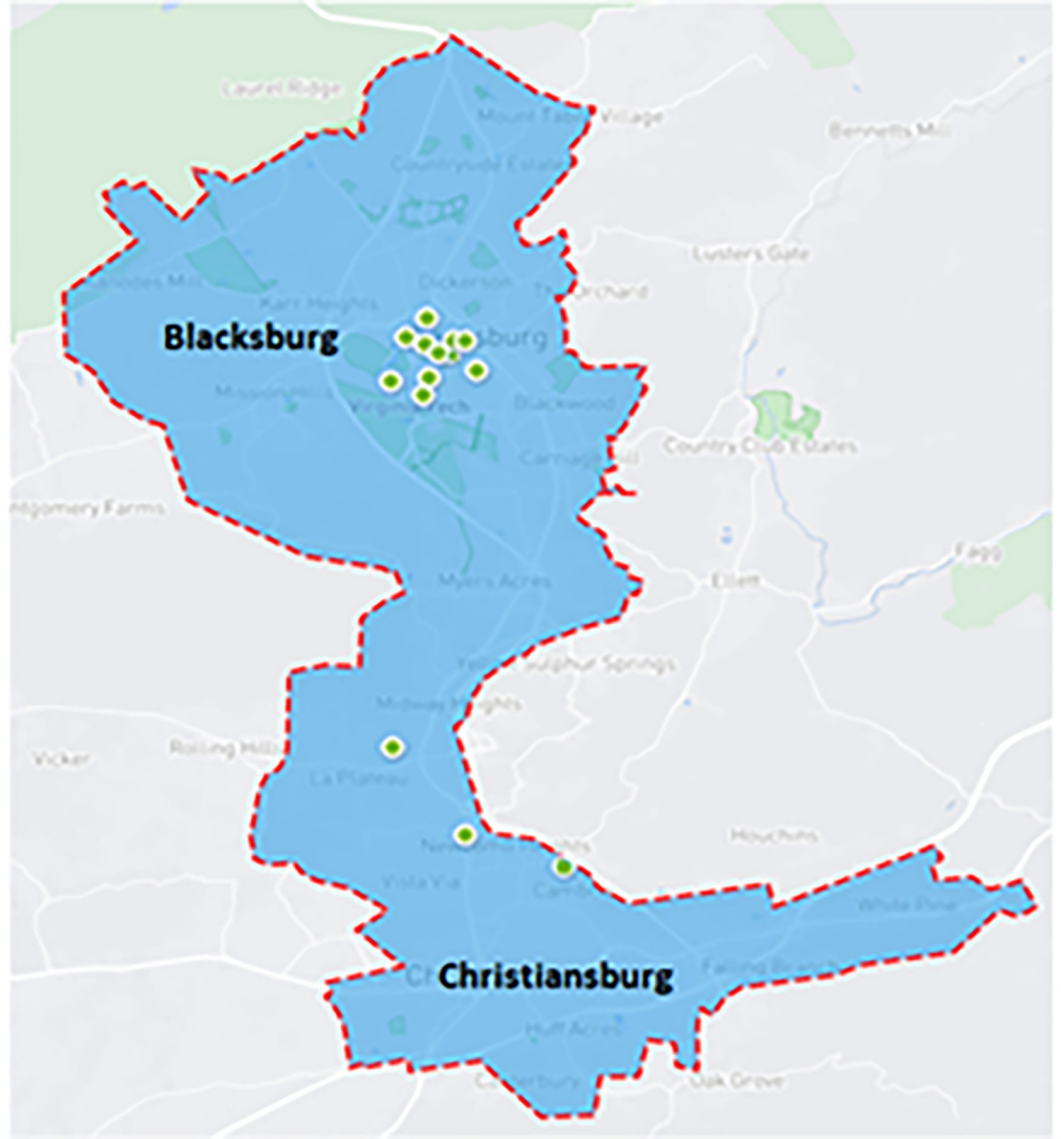
The boundaries of the bike sharing systems in Blacksburg and Christiansburg.

The study used data from a bike-sharing program in Montgomery County, Virginia, in the United States. Blacksburg and Christiansburg are the two towns it covers in the dataset used. It was established via the joint efforts of these two communities, the county, and Virginia Tech, and was advertised as (ROAM NRV). It was previously run by Gotcha Mobility LLC, which Bolt Mobility later purchased in 2021. Fig 1 illustrates the system’s initial launch in July 2018 with 12 bike stations and 75 bikes. Despite being a regional program, the system primarily serves the Virginia Tech community. The bike stations were distributed in the area as follows: The Town of Blacksburg (2 stations), The Town of Christiansburg (2 stations), and Virginia Tech Campus (8 stations). Since then, the system has expanded to include more of the New River Valley. The entire system was changed over to an electric-assist bike system in June 2021 with the same number of bikes but electrical ones (i.e. the fleet size remained constant).

**Fig 2:**
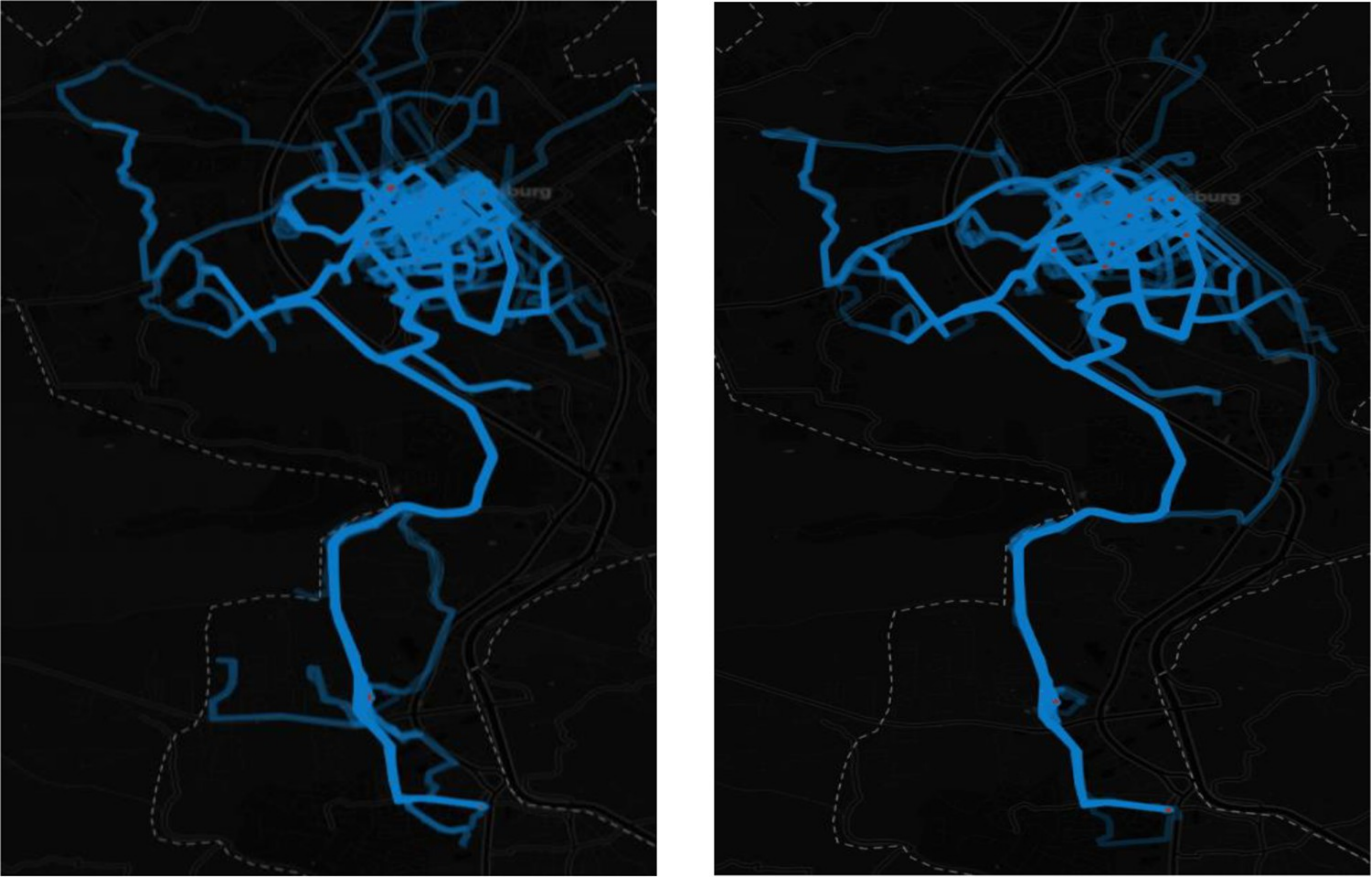
Heatmap of bikeshare users’ bike ride routes for June of 2020 and 2021 (left to right).

The information used includes statistics for both pedal and electric bikes from Jan 2019 to December 2021. During the study period, more than 14,555 trips totaling about 60,000 miles and 9,000 hours were made. Since its introduction, there have been 5154 active users, or users who have made more than one trip. The dataset captures information about each trip made, including the name of the origin and destination, the path of the trip (visually represented by heat maps in Fig 2), the start and end times of the trip, the miles traveled, the duration in minutes, the calories burned, the type of trip (unique or regular), the type of user (active or non-active), and whether the user is new or returning.

### Research framework and model approach

The system automatically calculates the calories burned for each trip made. It estimates the number of calories for a pre-defined average person based on distance traveled, while the carbon reduced is calculated by estimating the amount of Carbon Dioxide (CO2) saved over driving in a vehicle. The system classifies trips to either unique or regular based on the beginning and ending of the trip. It is a regular trip if taken and returned from/to a designated hub with no lock period or holds, while it is considered a unique trip if otherwise (i.e. unlocking of a bike, riding to a destination, locking the bike then unlocking it again and continuing to the completion of the ride at a designated hub). The trip length is calculated automatically in minutes for the period between unlocking and locking again the bike.

In this research, we will use five parameters for the analysis, namely: the number of calories burned, the amount of Carbon Dioxide (CO2) saved, the trip length and duration, and the type of the trip.

### Blacksburg transit

The local public transportation system was significantly affected by changes to its operating service, fare prices, and necessary Centers for Disease Control and Prevention health protocols for both its drivers and passengers. Blacksburg Transit first cut the number of its routes and its frequency. It also introduced bus passenger capacity restrictions, which limited large bus lines to a maximum of nine people and their smaller vans to a maximum of five or six. During the first year of the epidemic, Christiansburg’s routes and services were completely cut off. Prior to COVID, people having a university ID (Hokie Passport) could ride for free, however since the pandemic, all transit users can ride for free. Ridership for public transportation was considerably impacted negatively by these developments, combined with worries about safety and health. A rise in the use of active transportation, including walking and biking and the usage of bikeshare, was seen as a result of the decline in bus ridership.

## Results and discussion

A descriptive analysis for the ROAM NRV system’s usage was made using more than 14,555 trips totaling about 60,000 miles and 9,000 hours during Jan 2019 to December 2021, covering the COVID-19 pandemic. A summary of COVID-19 timeline for the Montgomery county is given in Table 1. For a more in depth look at the COVID-19 timeline in Virginia, refer to S1 Appendix.

**Table 1:** An overview of COVID-19 orders and restrictions in Virginia (as of August 3, 2020).

Stay at home effective	Stay at home relaxed	Travel permitted outside the home	Social Gatherings	Businesses reopened	Quarantines	Restaurants reopened	Beaches/ parks reopened
30-Mar 2020	10-Jun 2020	Essential needs/work only	10 person limit	With limitations	No directives	Dine-out and outdoor only	Limited access

During the COVID-19 pandemic, the operator of ROAM NRV had resiliency measures and efforts to protect the riders and keep the system safe, as follows:

- Submitting COVID-19 protocols to the partners of the system; Town of Blacksburg (TOB), Town of Christiansburg (TOC), Montgomery County and Virginia Tech (VT)
- Issuing communications describing the safety precautions being implemented
- Introducing personal protective equipment (PPE) and other measures for employee protection
- Properly washing/sanitizing hands (according to Centers for Disease Control (CDC) protocols)
- Socially distancing in the warehouses
- Increasing cleaning and sanitation of bikes and stations (using CDC approved cleaning practices and supplies)

However, while the ROAM NRV bikeshare maintained uninterrupted service during this time, it did experience a slight drop in overall ridership 2019–20 (down 3.56% in new member sign ups), fortunately for the bikeshare system, this was short lived with ridership experiencing an upward trend in 2021 (up 11.39% in new member sign ups). This bikeshare also underwent a complete system change in June 2021, converting its bike fleet from pedal bikes to electric assist, and changing the app platform users’ interface with. These dramatic changes also played a role in the jump in ridership as seen in Table 2.

**Table 2:** Monthly breakdown of ridership based on new signup.

	Jan	Feb	Mar	Apr	May	Jun	Jul	Aug	Sep	Oct	Nov	Dec
**2019 (pedal)**	30	82	175	262	346	193	229	217	325	145	34	43
**2020 (pedal)**	26	46	220	194	316	218	217	236	230	163	105	35
**2021 (pedal/e-bikes)**	24	42	158	215	215	367	397	372	270	225	97	79

### Trip trends and patterns

**Fig 3:**
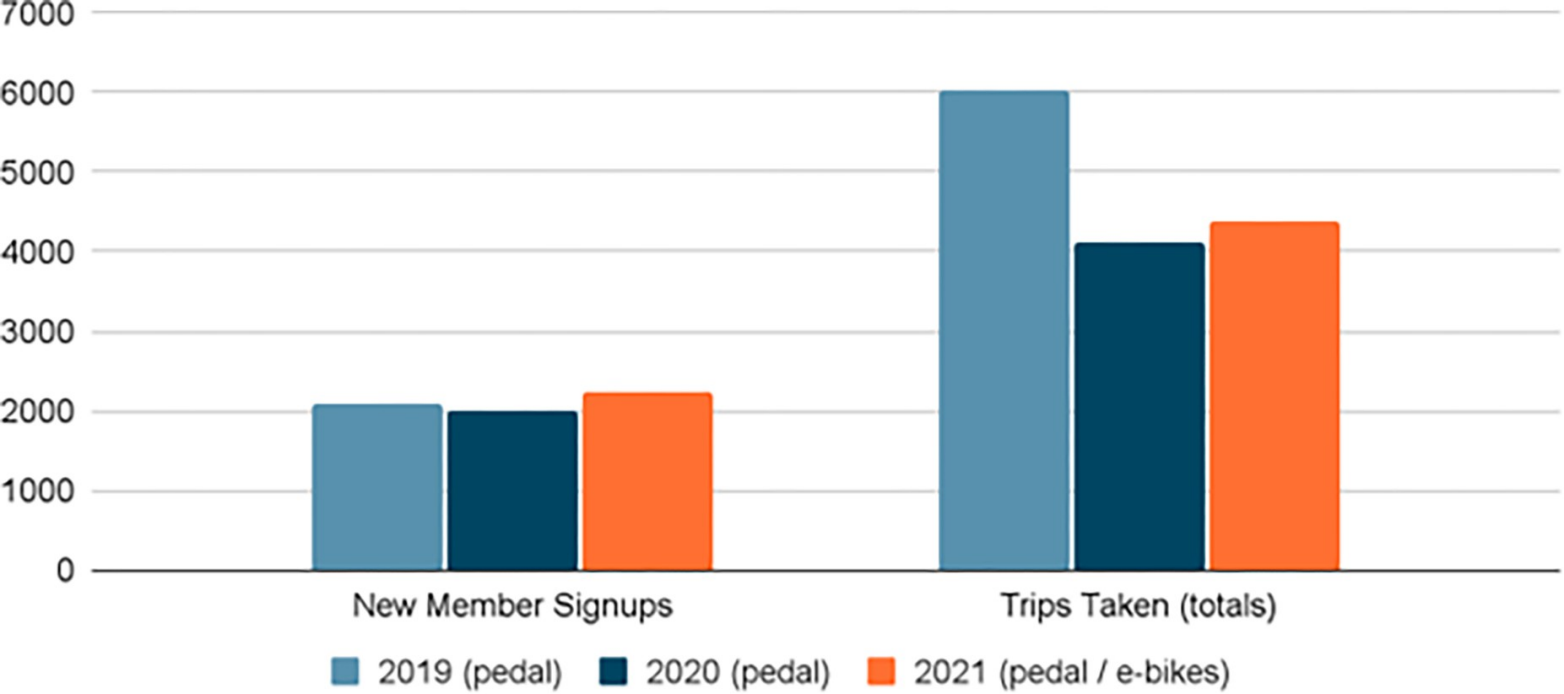
Annual ridership (memberships + trip activity), 2019–2021.

**Fig 4:**
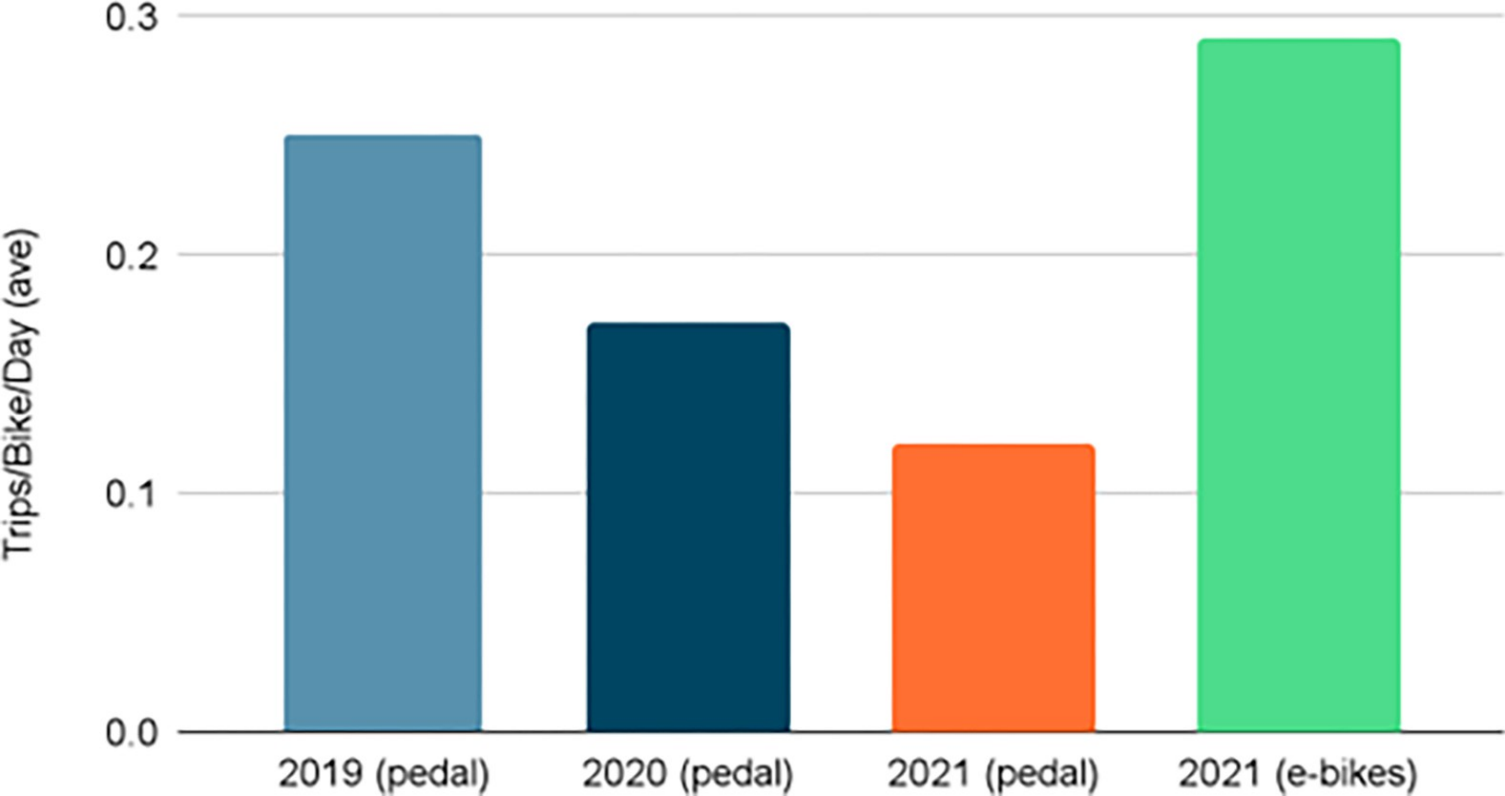
Annual bikeshare system utilization, 2019–2021.

As seen in Fig 3, approximately 2,075 Montgomery County residents and visitors took over 6,000 trips on 75 bikeshare pedal bikes in 2019, while 2,001 residents and visitors took over 4,100 trips on those same 75 pedal bikes the following year. This was about two thirds of the trips taken in 2019. However, bikeshare membership was at its highest in 2021, surpassing pre-COVID numbers and the system’s utilization (measured by the number of trips taken per bike per day) also increased, more than doubling (Fig 4) after the arrival of e-bikes.

**Fig 5:**
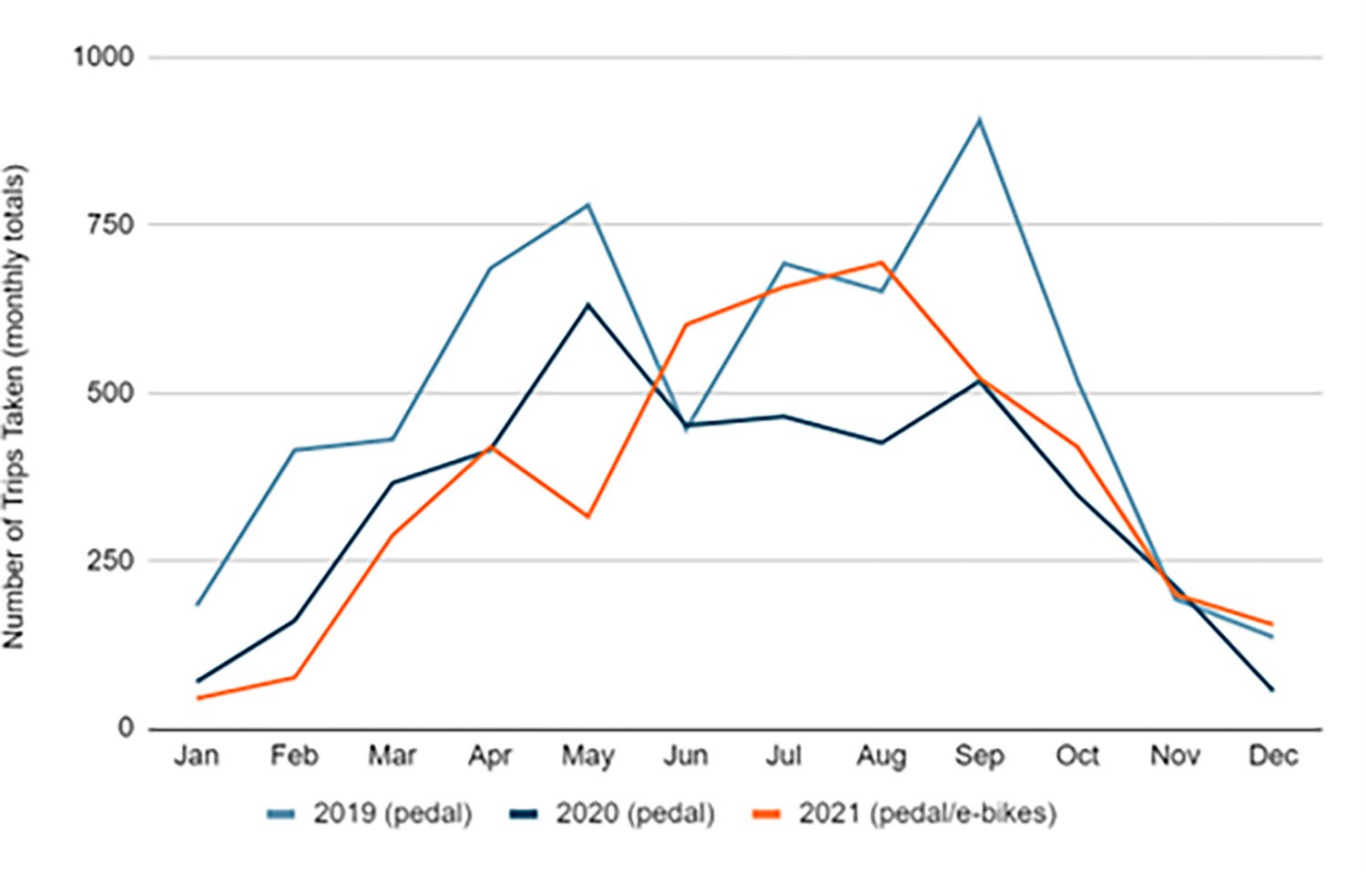
Monthly trip activity, 2019–2021.

**Fig 6:**
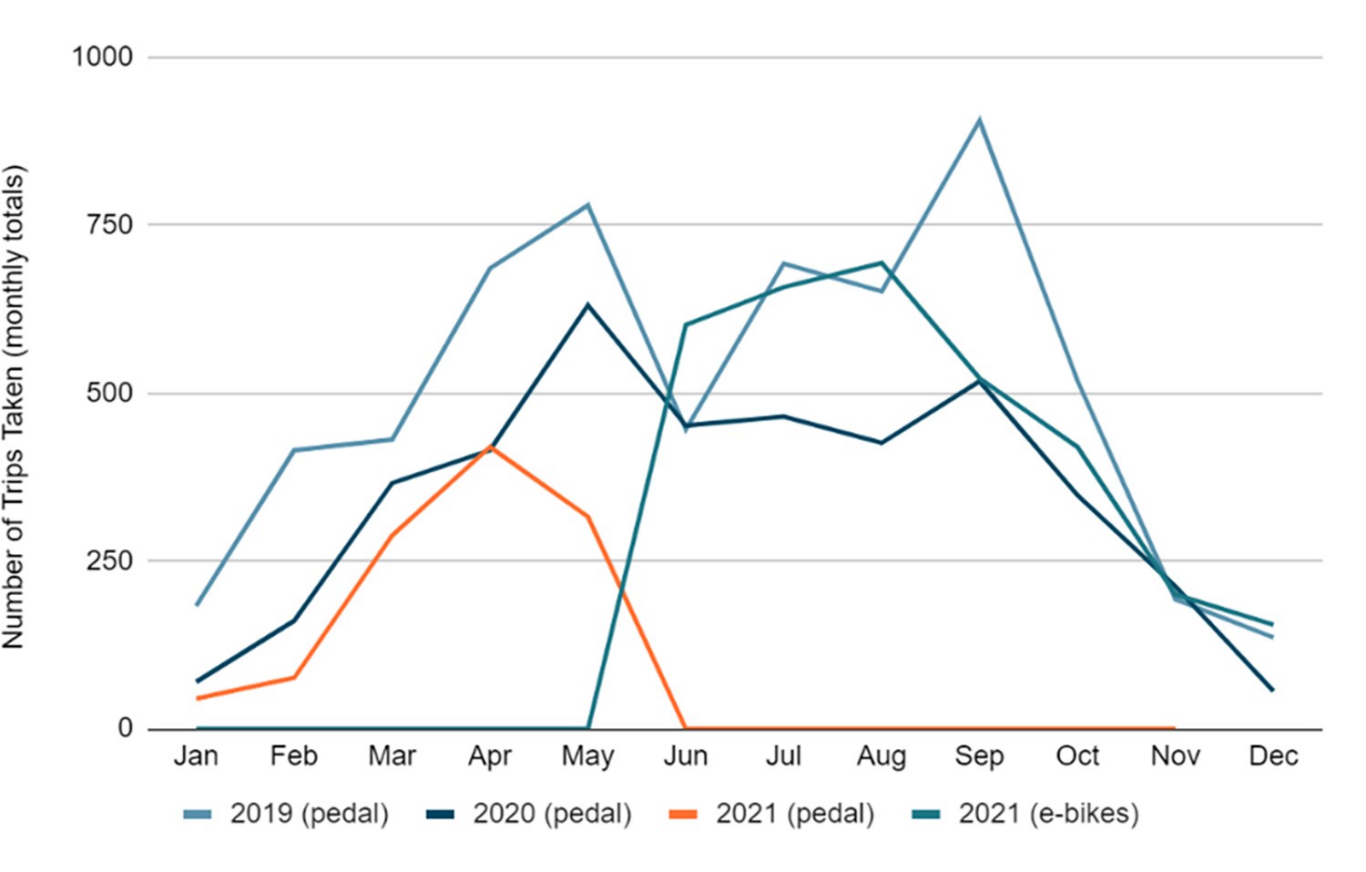
Monthly trip activity: Pedal VS E-bikes, 2019–2021.

Fig 5 shows the number of monthly trips taken between 2019 and 2021. Generally, the winter months had the least demand in the year, while the summer months (April- Aug) had the highest demand. We can also notice that there is a clear drop in April 2020, compared to the same month in the previous year due to the spread of COVID-19 as travelers try to avoid getting out of home. Interestingly, the total number of trips for the following month (May 2020) had the highest trip in the 2020 year although this month falls into the COVID-19 restrictive period as shown in Table 2. This can be justified that people find the bikeshare a safe transport mode to commute in the county. In 2021, monthly trips had jumped dramatically in June due to replacing the conventional bikes with e-bikes. Fig 6 makes a comparison of the number of monthly trips taken by pedal and e-bikes in the same period (2019–2021).

**Fig 7:**
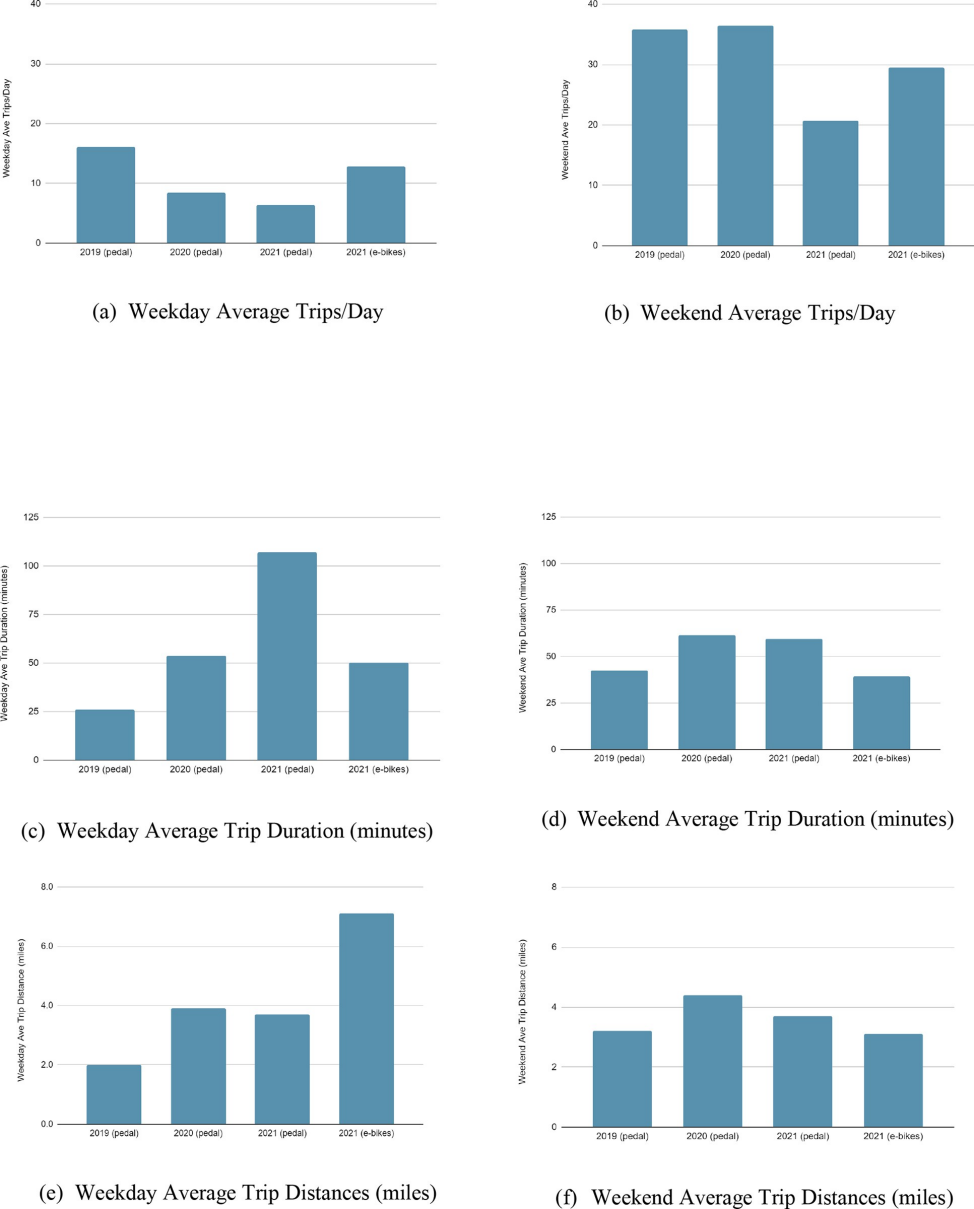
Annual average trips, duration and distances: Weekday VS Weekend, 2019–2021.

As shown in Fig 7, a bikeshare user’s average trip distance and duration (how long a trip took, measured as minutes per trip) increased, nearly doubling 2019–20 from 2+ miles to 4+ and from half an hour to about an hour respectively. While there was a slight drop in 2021 for both, bikeshare users continued to travel farther distances and spend more time on the bikes than pre-COVID trips. This result agrees with a similar study conducted in London [16]. When those averages were unpacked to compare weekday trips to weekend trips, a few interesting trip patterns were observed. Unsurprisingly, more trips still took place on the weekends (increasing from 2x as many trips to 4x as many trips than the weekday). Weekday trips experienced a declining trend during the pandemic only doubling after the arrival of the electric-assist bicycles. On the other hand, the average duration nearly doubled (~50 min) from 2020 to 2021 for pedal bikes, but dropping back to ~50 minutes with the e-bikes. Meanwhile, the average trip distance remained steady, most trips taken were less than 4 miles, then substantially increasing to more than 7 miles with the e-bikes (Fig 7C–7F). Weekend trips remained steady during the height of the pandemic only to drop a bit in 2021, until the e-bikes. The average duration of weekend trips remained relatively steady until the e-bikes in which case average minutes per trip dropped, likely due to electric bikes having the capacity to travel faster, meaning a rider on an e-bike would take less time to travel the same distance as someone on a pedal bike. A similar pattern emerges with trip distance, however, where the anticipated distance traveled was expected to increase as well, it dropped.

**Fig 8:**
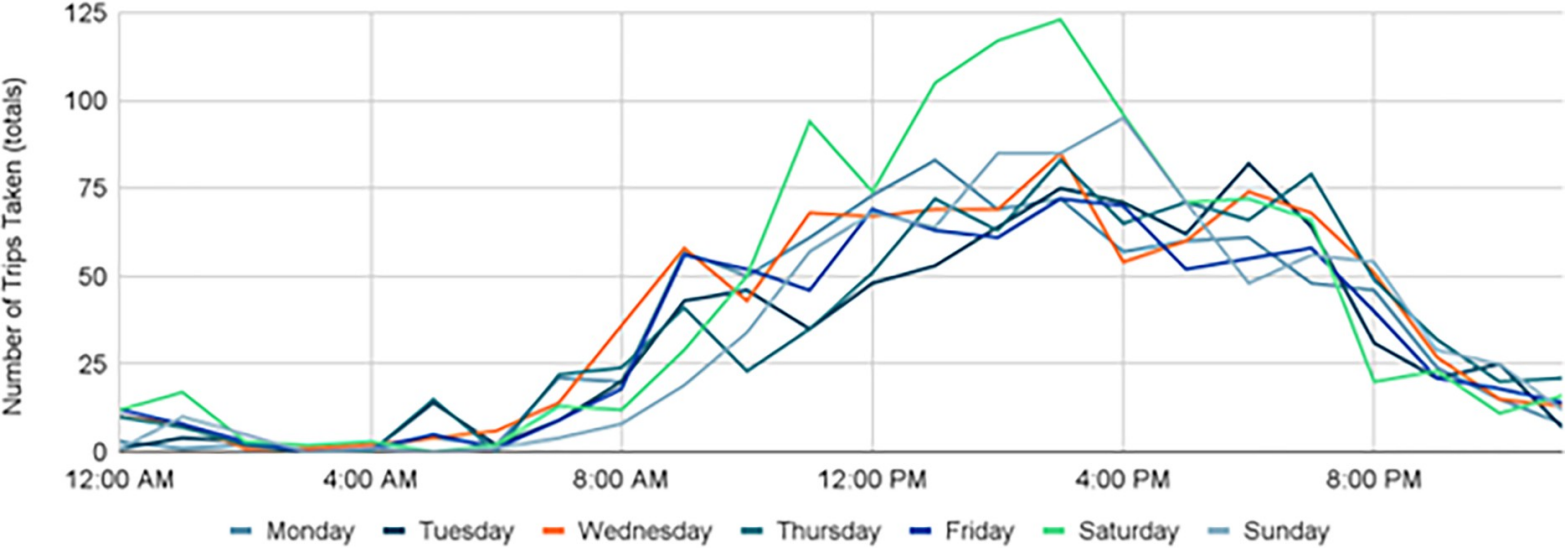
Trips by time of day, 2019.

**Fig 9:**
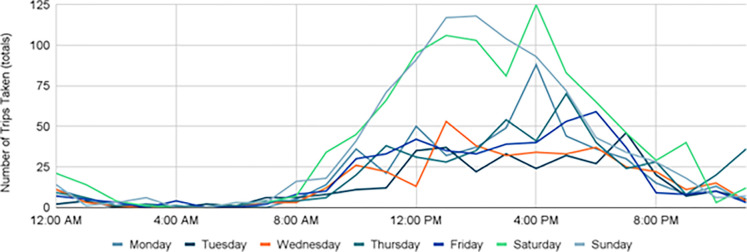
Trips by time of day, 2020.

**Fig 10:**
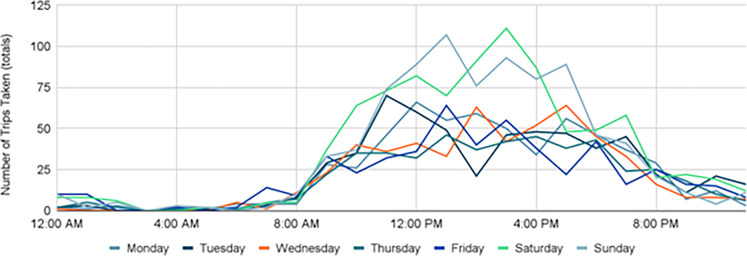
Trips by time of day, 2021.

Overall, peak travel times remained constant 2019–2021, with the highest number of trips taking place around 2–3 PM, but a slight change in morning and evening times did occur. Pre-COVID, trips typically took place between 9AM - 7PM with Saturdays experiencing the greatest amount of trip activity (Fig 8). During the height of COVID-19, that shifted to later in the day with trips typically occurring from 12–6 PM, with Saturdays and Sundays experiencing the greatest amount of trip activity (Fig 9). This was likely due to Stay-at-Home orders placed early on, and many residents that transitioned to remote work and class environments continued even after the order was lifted. As more restrictions were lifted and campus returned to in-person classes (with hybrid options), trips returned to their pre-COVID travel hours between 9AM - 7PM while Saturdays and Sundays remained steady with high trip activity (Fig 10).

### Population

According to the 2020 census, the total population for Montgomery County VA was 99,721 residents (1.61% increase from 2019) with 44,826 residing in the Town of Blacksburg (1.18% increase from 2019) and 22,163 residing in the Town of Christiansburg (1.48% increase from 2019). About 83% of the Town of Blacksburg community comprises the Virginia Tech student population. The university’s student body was made up of 37,024 students for the academic year 2020–2021 (0.04% increase from academic year 2019–2020). During the height of the pandemic, population size dropped 0.52% for the Town of Blacksburg, and increased 1.15% for the Town of Christiansburg. A notable impact for the university was its pivot to a virtual classroom environment 2020–2021, which returned to an in-person class environment with hybrid options the following year. Additionally, residential halls that had closed in 2020 started reopening in 2021, allowing more students to live on campus again, which led to an 11.96% spike in the student population.

Upon observing the heat maps of bikeshare users (Fig 2), the primary area of for ride concentration remained on and around the university’s campus 2020–21. This is likely because 2/3rds of the area’s population and a disproportionate number (~70%) of the system’s stations are located there. This remained true, even as the campus (and town) went into lockdown and students had to move off campus and/or back home. However, campus-based trips (to/from academic buildings, dining halls, on-campus housing, and other campus facilities) did drop, users also traveled more into their surrounding community off-campus and an increased use of the trail system, particular the local Huckleberry Trail network, was noted.

It is worth mentioning that while vaccine mandates have been in place for students, as a prerequisite to returning to campus, and for faculty and staff members, and much of the surrounding community and businesses have reopened, the long-term impacts are still taking place. For bikeshare operators in particular the long term and possible permanent effects of supply chain issues, manufacturing and shipping delays are still unknown.

### Rural residents benefit from bikeshare

**Fig 11:**
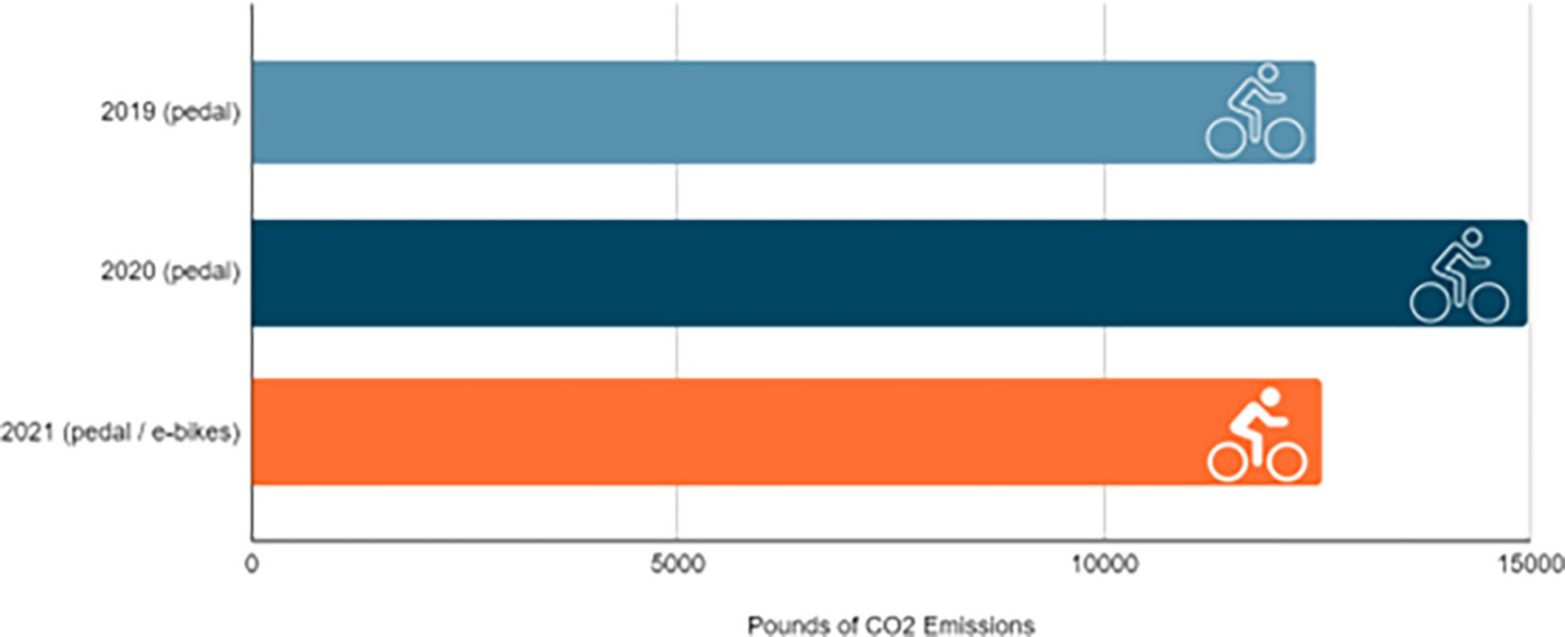
Annual amount of carbon emission saved (in pounds) 2019–2021.

Notable benefits of bikeshare include the decreased street congestion which leads to less carbon emission output, shorter commute times for some, and a healthy alternative to driving. Commuting by bikeshare produces considerably fewer greenhouse gas emissions (GHG), reducing GHGs by 100% on pedal bikes and 97% on e-bikes, compared to auto trips [17]. ROAM NRV bikeshare users offset nearly 12,500 pounds of carbon emissions in 2019, ~15,000 pounds in 2020 (~2,500 more than 2019) and more than 12,500 pounds (3,740 via pedal bikes and 8,776 via e-bikes) in 2021, just by replacing auto trips with pedal and e-bike trips (Fig 11).

**Fig 12:**
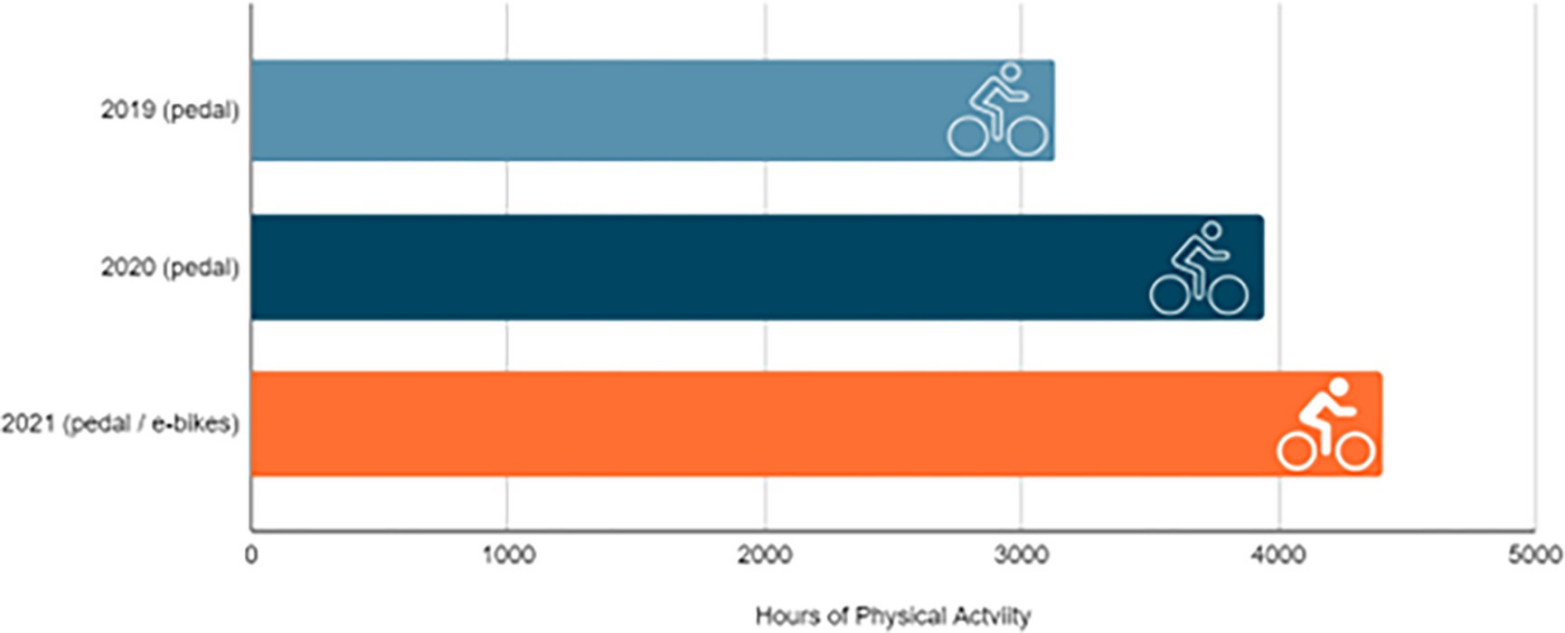
Annual hours of physical activity via bikeshare 2019–2021.

Exercise is an essential component of a healthy lifestyle. Moderate exercise like pedal and e-biking have powerful, measurable effects on health. In 2019, bikeshare users gained 3,130 hours of physical activity, collectively burning 566,010 calories and nearly 4,000 hours of physical activity (over 800 more than 2019) (Fig 11), collectively burning 677,712 calories (Fig 12) through bikeshare use alone. This indicated that residents and visitors using the bikesharing system were more active during the pandemic than previously. As new Coronavirus variants emerged, additional precautions and new protocols went into effect during 2021. Despite this, residents and visitors were still getting a combined effort of nearly 4,400 hours of additional physical activity (1,679.4 hours on pedal bikes and 2,715.07 hours on e-bikes) in 2021. Those using pedal bikes during this time, collectively burned 169,685 calories. It is worth noting that after switching to e-bikes, caloric data was unavailable but likely lower due to the assistance the e-bike provides, taking on more of the workload.

## Conclusion

Rural Americans faced unique transportation challenges before COVID-19 and as a result of the pandemic, those challenges are further exacerbated, particularly at providing equitable active transportation. New mobility options, like Bikesharing systems, offer cost-effective solutions that can complement higher-volume, fixed-route transit services and address other challenges such as safe and affordable transportation, changing rural populations, and higher health risk concerns. Bikeshare can also expand the reach of traditional public transit systems with its first-last mile connections. Bikeshare emerged as a new mobility option in Montgomery County, VA in 2018, and has remained resilient throughout the pandemic. Despite the initial decline in ridership, and given the added benefit of e-bikes, ridership started growing again. Demand has also gradually started to pick up from its pre-COVID-19 levels.

In this research, we aim mainly at investigating the effect of COVID-19 on bike share usage in rural areas, particularly, Montgomery County area. Thus, spatial and temporal analysis of trips made from 2019 to 2021, covering three periods: before, during-, and post-pandemic. Behavior change was noticeable during the timespan of the study, including longer and farther trips patterns. Interestingly, the monthly trips for the May 2020 had the highest trips in the 2020 year although this month falls into the COVID-19 restrictive period. This proves people used bikeshare as a safe transport mode during the pandemic. Results also show weekend trips remained steady during the height of the pandemic only to drop a bit in 2021, until the e-bikes.

We also investigated the effect of e-bikes added to the system and found riders had increased the trip’s distance but reduced tits duration. The speed and added assistance up hills e-bikes offer, is likely one determining factor for users to choose bikeshare to ride more often, longer distances while saving time. Additionally, as more people have returned to the office and campus, and Blacksburg Transit has mostly returned to its pre-COVID operations (masks still required), bikeshare continues to offer a mobility option for people still hesitant to use public transportation, but still care about the environmental impacts of driving, want to maintain healthy habits that they may have gained or increased during the pandemic, and/or have fewer mobility options than those with private vehicle access.

To the best of our knowledge, this study is the first to investigate the bike’s usage in a small rural community considering five parameters, namely: the number of calories burned, the amount of Carbon Dioxide (CO_2_) saved, the trip length and duration, and the type of the trip, in a rural small community. We believe that this study sheds the light on important insights for using bikesharing systems in rural areas and small communities such as Blacksburg and Christiansburg. The study is beneficial for policymakers and researchers as it brings new understanding of bikesharing systems in rural areas. However, for future study, considering the demographic characteristics of the users and types of the trips will be beneficial to further enhance the analysis of this study.

## Supporting information

S1 Appendix (DOCX)

## References

[1] Henning-Smith C., et al., Rural transportation: Challenges and opportunities. Policy Brief. University of Minnesota Rural Health Research Center. http://rhrc.umn.edu/wp-content/files_mf/1518734252UMRHRCTransportationChallenges.pdf, 2017.

[2] Officials N.A.o.C.T., 136 Million Trips Taken on Shared Bikes and Scooters Across the U.S. in 2019. 2020.

[3] Statistics B.o.T. Bikeshare Ridership Down 44% During COVID-19. 2020 [cited Aug. 2022; Available from: https://www.bts.gov/newsroom/bikeshare-ridership-down-44-during-covid-19.

[4] Statistics B.o.T. COVID-19 Crushes Bikeshare and E-scooter Ridership, Closes Systems Permanently in Some Cities. 2021 Aug 2022]; Available from: https://www.bts.dot.gov/data-spotlight/covid-19-crushes-bikeshare-e-scooter-ridership.

[5] Statistics B.o.T. COVID-Affected Micromobility Changes Differ by City. 2021 Aug 2022]; Available from: https://www.bts.dot.gov/data-spotlight/covid-affected-micromobility-changes-differ-city.

[6] Transportation B.o. Effects of COVID-19 on Bikeshare (Docked and Dockless) and E-scooter Operation. 2022 Aug 2022]; Available from: https://data.bts.gov/stories/s/Effects-of-COVID-19-on-Bikeshare-Docked-and-Dockle/kar5-6dpn/#:~:text=In%20summary%2C,throughout%20March%20to%20December%202020.

[7] Statistics B.o.T. Bikeshare and E-scooter Systems in the U.S. 2022 Aug 2022]; Available from: https://www.bts.gov/topics/passenger-travel/bikeshare-and-e-scooters.

[8] Campisi T., et al., The impact of COVID-19 pandemic on the resilience of sustainable mobility in Sicily. Sustainability, 2020. 12(21): p. 8829.

[9] Almannaa M.H., et al., Perception analysis of E-scooter riders and non-riders in Riyadh, Saudi Arabia: Survey outputs. Sustainability, 2021. 13(2): p. 863.

[10] Mouratidis K., How COVID-19 reshaped quality of life in cities: A synthesis and implications for urban planning. Land Use Policy, 2021. 111: p. 105772. 34566233 10.1016/j.landusepol.2021.105772 PMC8456312

[11] Nikitas A., et al., Cycling in the era of COVID-19: Lessons learnt and best practice policy recommendations for a more bike-centric future. Sustainability, 2021. 13(9): p. 4620. doi: 10.3390/su13094620

[12] Menon N., Keita Y., and Bertini R.L., Impact of COVID-19 on Travel Behavior and Shared Mobility Systems. 2020.

[13] Today U. Study finds coronavirus safety communication matters for bike share ridership. 2021 Aug 2022]; Available from: https://www.utsa.edu/today/2021/03/story/griffin-jobe-communication-bike-sharing.html.

[14] Padmanabhan V., et al., COVID-19 effects on shared-biking in New York, Boston, and Chicago. Transportation research interdisciplinary perspectives, 2021. 9: p. 100282. doi: 10.1016/j.trip.2020.100282 33748743 PMC7964246

[15] Rahman S.M., et al., Transformation of urban mobility during COVID-19 pandemic–Lessons for transportation planning. Journal of Transport & Health, 2021. 23: p. 101257. 34580629 10.1016/j.jth.2021.101257 PMC8459165

[16] Heydari S., Konstantinoudis G., and Behsoodi A.W., Effect of the COVID-19 pandemic on bike-sharing demand and hire time: Evidence from Santander Cycles in London. PloS one, 2021. 16(12): p. e0260969. 34855914 10.1371/journal.pone.0260969 PMC8639062

[17] NABSA, 2019 NABSA 1ST ANNUAL Shared Micromobility State of the Industry Report. 2021.

