# Supplementary material for: The COVID-19 impacts on bikeshare systems in small rural communities: Case study of bikeshare riders in Montgomery County, VA
Source: PLoS One. 2022 Dec 1;17(12):e0278207. doi: 10.1371/journal.pone.0278207 (PMC9714740; doi:10.1371/journal.pone.0278207)
Supplement: S1 Appendix — (DOCX) [file pone.0278207.s001.docx]

**SUPPORTING INFORMATION**

**APPENDIX A**

| **2020** | COVID-19 Timeline in Virginia |
| --- | --- |
| March 7 | First COVID-19 case in VA |
| March 11 | W.H.O declares COVID-19 a worldwide pandemic |
| March 12 | VA Governor Northam declares a state of emergency |
| March 13 | US declares COVID-19 a national emergency |
| March 14 | First COVID-19 death in VA |
| March 23 | VA public schools close. VA businesses (bowling alleys, gyms, theaters) close |
| March 24 | VA restaurants close dining rooms. Delivery and takeout service remained available |
| March 30 | Governor Northam issues statewide Stay at Home order. VA colleges and universities stop in-person classes |
| April 6 | 90% of US population under social distance orders |
| May | Social distancing orders relaxed in most states |
| May 15 | Most of VA enters Phase One reopening |
| May 29 | Masks required in public indoor spaces in VA |
| June 5 | Most of VA enters Phase Two reopening |
| July 1 | Most of VA enters Phase Three reopening |
| Nov 15 | VA implements new COVID-19 restrictions |
| Dec 14 | Stay at Home orders between 12-5AM, universal mask requirement and limit to 10 people at social gatherings implemented. First shipments of vaccine arrive in VA |
| **2021** |  |
| March 1 | Outdoor gathering restrictions ease - limit of people at social gatherings increased to 25. Capacity restrictions increased at outdoor entertainment venues. |
| April 1 | VA allows certain sports and entertainment venues to increase capacity |
| April 29 | Fully-vaccinated Virginians no longer required to wear masks outdoors when alone or in small gatherings |
| May 14 | VA lifts universal indoor mask mandate |
| May 28 | VA lifts social distancing and venue capacity restrictions |
| August | Virginia Tech reinstates in-person classes with hybrid option across campus locations |
